# An Overview of Missed Nursing Care and Its Predictors in Saudi Arabia: A Cross-Sectional Study

**DOI:** 10.1155/2022/4971890

**Published:** 2022-10-12

**Authors:** Essa H. Al Muharraq, Sultan M. Alallah, Saad A. Alkhayrat, Ali G. Jahlan

**Affiliations:** ^1^Nursing Administration Department, Al Darb General Hospital, Jazan Health Affairs, Jazan, Saudi Arabia; ^2^Nursing Education Department, Prince Mohammed Bin Nasser Hospital, Jazan Health Affairs, Jazan, Saudi Arabia; ^3^Regional Nursing Administration, Jazan Health Affairs, Jazan, Saudi Arabia

## Abstract

**Background:**

Nursing care is holistic, and missing any aspect of care can be critical to patients' health. However, due to the complex and intense nature of the nursing practice, nurses have to unintentionally prioritize some activities, forcing them to omit some aspects of nursing care.

**Aim:**

To explore the dimensions of missed nursing care and its predictors within the Saudi Arabian healthcare system.

**Methods:**

Quantitative, cross-sectional study used the MISSCARE survey by utilizing nonprobability convenience sampling to collect the data of 604 staff nurses working in inpatient wards in Jazan, Saudi Arabia.

**Results:**

The overall mean of missed nursing care is (*m* = 1.37, SD = 0.45). Missed nursing care activities were mostly failure to attend interdisciplinary care conferences (*m* = 1.66, SD = 0.96) and patient ambulation thrice a day (*m* = 1.63, SD = 0.97). Missed nursing care was mainly caused by human resource shortage (*m* = 3.53, SD = 0.88). Missed nursing care is predicted by the turnover intention (*B* = 2.380, *t* = 3.829, *p* < 001) and job satisfaction (*B* = −0.864, *t* = −4.788, *p* < 001).

**Conclusion:**

Although missed nursing care is evident in Saudi Arabia, it is significantly lower than the international rates, and it is mainly caused by labor resource shortage which directly influences nurses' job satisfaction and intention to leave. Optimizing the recruitment process, resource allocation and effective nurses' retention programs are proposed solutions that may be beneficial to mitigate missed nursing care.

## 1. Introduction

Ensuring optimum care and safe practice is the core objective of all healthcare organizations. The World Health Organization (WHO) defines patient safety as the prevention or mitigation of errors and harms to patients associated with the provision of health care (2017). The Institution of Medicine encourages surveillance and reporting of errors in healthcare, with several initiatives, to measure and mitigate errors [1]. These errors are classified into two major types: errors of the commission as a result of the wrong action taken and errors of omission as a result of actions not taken or missed [2].

Nurses play a crucial role in preserving patient safety [3], considering that they constitute the largest portion of healthcare providers [4]. They also play an active role in detecting and preventing errors [5]. Furthermore, the holistic merit of nursing practice demands direct interaction and intervention with patients. This complex and intense nature of nursing practice make nurses prone to committing unintentional errors [6], and as a result of time constraint and insufficient manpower and resources, they often have to consciously prioritize some activities over others [7].

Missed nursing care is an omission error wherein some aspects of required nursing care are partially or totally delayed or unfulfilled [8]. It occurs in all cultures and countries [9] and is an important indicator of nursing care quality and patient safety [10]. This phenomenon has several names that are used interchangeably. Among them are missed nursing care [11], implicitly rationed care [12, 13] and nursing care left undone [14, 15]. Nevertheless, all of them denote the omission or delay of parts of nursing care [16].

A pioneer qualitative study [11] interviewed nurses to determine the frequency and reasons behind missed nursing care and reported the following nine major elements of nursing care that are usually missed: ambulation, position change, feeding, patient teaching, discharge planning, emotional support, hygiene, intake, and output documentation. According to Kalisch et al. [9], missed nursing care is an omission error in which its antecedents include labor resources, material resources, and communication/teamwork, with serious consequences and threats to patient safety.

However, factors associated with missed nursing care still remain poorly explored. Ausserhofer et al. [14] reported that the frequency of missed nursing care is clearly associated with both the nursing work environment and the nurse-to-patient ratio. Working under pressure with time and resource restrictions leads nurses to commit errors of omission and neglect of care [17]. Kalisch [11] enumerated different reasons behind missed nursing care; among them are staffing inadequacy, time restriction, poor delegation, and insufficient material resources. In 2021, Al-Fauri, Obaidat, and AbuAlRub explored the types and reasons for missed nursing care among Jordanian nurses and inferred that inadequate manpower is the most frequent cause of missed nursing care and that a low nursing staffing ratio is associated with high missed nursing care. Furthermore, Alsubhi et al. [18] conducted an extensive review to identify factors associated with missed nursing care and nurses' voluntarily turnover; they found that missed nursing care is highly prevalent in many countries and is linked to negative patient outcomes, poor nursing satisfaction, and a high tendency for turnover.

Moreover, missed nursing care has detrimental consequences for both patients and healthcare organizations, and it is associated with poor nursing care, job dissatisfaction, absenteeism [7], poor retention, and staff morale [19]. Most importantly, it has a negative effect on patient safety [20, 21]. It leads to increased length of hospital stay, undesirable complications (e.g., bed sores, falls, and hospital-acquired infection) [22], ineffective pain management, malnourishment, and high mortality [23].

In Saudi Arabia, few studies explored missed nursing care and mainly focused on its relationship with the practice environment and safety. To the best of our knowledge missed nursing care and its predictors in Saudi Arabia have not yet been investigated. Hence, this study aimed to explore the most common types and reasons behind missed nursing care and its predictors in a unique multinational nursing workforce in Saudi Arabia.

## 2. Methods

### 2.1. Design

This cross-sectional, observational, quantitative study explored the most common types and reasons for missed nursing care and its predictors.

### 2.2. Settings

The study was conducted among nurses working in inpatient wards in the Jazan area (2 tertiary and 8 general hospitals) with 1565 bed capacity and 1566 nurses working as staff nurses in inpatient wards in total.

### 2.3. Participants and Sampling

Data were collected from participating nurses between June 1, 2021, and September 30, 2021. As COVID-19 pandemic precautions demand physical distancing, the study used an online data collection method. The study questionnaire was created using Google Forms, and the link was shared with the regional nursing administration in Jazan and consecutively forwarded to Directors of Nursing in the proposed hospitals, and they distributed to their nurses. Convenience sampling was utilized. Inclusion criteria for participation required nurses to be working in inpatient wards and provide direct nursing care. Meanwhile, nurses working in the outpatient department, administration, and those who are not involved in providing direct nursing care were excluded. Yamane's sample size formula: *n* = *N*(1+N^*∗*^e^2^) [24], used to determine the appropriate sample size with 95% confidence level, 5% margin of error and study population of 1566, *n*= (319). Out of 1566 projected participants, only 604 participants completed the survey.

### 2.4. Study Questionnaire

The study employed the MISSCARE survey, which was developed by Kalisch and Williams in 2009. It consists of two main sections: the first section measures missed nursing care, which has 24 items. The participants were asked to indicate how often the nursing care is missed for each item, using a 5-point Likert scale ranging from never missed = 1 to always missed = 5 The second section of the MISSCARE survey which consists of 17 items aims to explore the reasons that drive nurses to MISSCARE. The survey requires participants to rate the reason for missed nursing care as significant, moderate, minor, or not a reason for missed nursing care. Participants' demographic characteristics, including age, gender, level of education, work experience, overtime, and working shifts were also included. The survey designates 3 items to measure nurses' job satisfaction which are the level of nurse satisfaction in the current position, the level of nurse satisfaction with being a nurse, and nurse satisfaction with the level of teamwork in the unit. In addition, nurses' intention to leave is measured by 1 item that asks participating nurses to report their intention to leave their current work within 6 months, within one year, or no intention to leave. In the present study, the study questionnaire was tested by 15 nurses in a pilot study to confirm the reliability and applicability, and Cronbach's *α* was 0.93 and 0.95 for the first and second sections, respectively, of the MISSCARE survey.

### 2.5. Ethical Considerations

Prior to data collection, permission to use the MISSCARE survey was granted by the original author. The electronic survey began with informed consent, all participants needed to read and chose to agree option to start filling out the questionnaire. The data collection method did not pose any health risk, and the participants' privacy was carefully preserved. Ultimately, to further enhance anonymity, the participants and their responses were coded so that their data could not be readily used for nonresearch purposes.

### 2.6. Statistical Analysis

All statistical data were analyzed using SPSS version 25.0. The data collected from the MISSCARE survey were analyzed using descriptive statistics, including mean and standard deviation (SD). Internal consistency of the study tool and the association of missed nursing care with job satisfaction and intention to leave were evaluated by correlational analysis including Pearson correlation coefficient and Spearman's rho correlation coefficient. Linear regression was used to test if variables can predict missed nursing care.

## 3. Results

Out of 1566 projected participants, the study recruited only 604 participants (response rate = 39%), 89.1% were female, 69.9% aged 25–34 years, and 51.3% had a bachelor's degree in nursing. In addition, 30.1% of the participants had been working for a period extended between 5 and 10 years. Meanwhile, 47% had been working overtime for 1–2 hours in the last 3 months, and 80.1% had no plans of leaving their current work (Table 1).

7.3% of participants reported that “attending interdisciplinary care conferences whenever held” was the most frequently missed element of nursing care (mean (*m*) = 1.66). Another element that was also frequently missed by 6.6% of participants was “facilitating ambulation thrice per day or as ordered” with *m* = 1.63). Conversely, “bedside glucose monitoring as ordered” recorded the least missed care activities as 90.7% of participants never missed (*m* = 1.12 (Table 2).

Table 3 summarizes the common reasons behind missed nursing care. The most frequently reported was “inadequate number of staff” (*m* = 3.53). This reason was reported to be significant. Other significant reasons were “inadequate number of assistive and/or clerical personnel (nursing assistants, technicians, and unit secretaries)” (*m* = 3.36), “unexpected rise in patient volume and/or acuity on the unit” (*m* = 3.29), and “heavy admission and discharge activity” (*m* = 3.15).

Correlation analysis was rendered between the study's main variables and missed nursing care. Missed nursing care had a significantly positive relationship with nurses' intention to leave (*r* = 0.199, *p* < 0.001) and a significantly negative relationship with nurses' job satisfaction (*r* = −0.297, *p* < 0.001) (Table 4).

Variables of age, gender, qualification, level of experience, adequate staffing, overtime, turnover intention, and level of satisfaction were tested if they predict missed nursing care among participants using multiple regression. Results show that 21% of the variance in missed nursing care can be accounted for by the seven predictors collectively F (10,593)= (16.319), *p* < 001. The results shows that the turnover intention (*B* = 2.380, *t* = 3.829, *p* < 001) and the level of satisfaction (*B* = −0.864, *t* = −4.788, *p* < 001) positively predict missed nursing care (see Table 5).

## 4. Discussion

Missed nursing care is a multifaceted phenomenon that has a direct impact on patients' health outcomes. This study aimed to determine the most common types and reasons behind missed nursing care and its predictors. The overall mean of missed nursing care in Saudi Arabia, as shown in this study (1.37), is significantly lower than those in the US (1.71) [25], South Korea (1.40) [26], and Australia (2.02) [27] as well as among middle eastern countries, such as Jordan (2.78) [28] and Egypt (2.26) [29].

The most frequently missed nursing care activities were failure to attend interdisciplinary care conferences, patient ambulation for three times a day, and turning and positioning every 2 hours, consistent with the results of several studies, including Gravlin and Bittner [30], Kalisch et al. [31], Papastavrou et al. [32], and Tubbs-Cooley et al. [33]. Nurses may perceive that these tasks are not a priority and that missing them will not cause serious impacts on patients' health. The most frequently missed task by nurses was attending interdisciplinary conferences, which can be attributed to work overload and lack of time to participate in such meetings [34]. In addition, nurses may probably have an unclear perception toward their role in the process of shared clinical decision and their contribution to the therapeutic process as a whole. Meanwhile, missing basic tasks including patient ambulation for three times and turning and positioning every 2 hours are often associated with the lack of supportive services and personnel such as aid nurses. These physical tasks require a certain level of strength and teamwork cooperation. Possibly, nurses missed these tasks because of their overdependence on family involvement in basic care. In Saudi Arabia, the healthcare system allows admitted patients to be accompanied by a family member to provide psychological support and ease the patients' hospitalization experience. Nurses may delegate under supervision some simple nursing tasks to patient attendants and request to assist in providing care, including ambulation, turning, and positioning.

The least common missed nursing care activities were bedside glucose monitoring and vital signs assessment. This result is consistent with several studies [10, 29, 31]. Nurses may perceive that such tasks are crucial and serious indicators of any deterioration in the general condition of patients and that they have a direct impact on patients' health outcomes. Nurses may also prioritize these tasks because it requires less time and effort to perform and needs precise documentation of results.

Furthermore, the most significant reasons for missed nursing care were inadequate number of staff, inadequate number of assistive personnel, patient volume/acuity, and heavy admission/discharge activity. Human resource shortage in hospitals is often regarded as one of the main factors of failure to provide comprehensive and quality care by nurses and nursing assistants. The gap between nursing workforce supply and demand reflects some serious local conditions. According to Aboshaiqah [35]; the limited number of nursing schools in Saudi Arabia, low wages, long working hours, and the negative social perception of nursing are the reasons why many nurses give up their nursing careers and why the country is experiencing an exacerbation of nurse shortage. These results suggest that the system of care in hospital units, where admissions and discharges and acuity fluctuations are the regular ebb and flow of the unit work, is not responsive to workload volume.

Although the Saudi Ministry of Health and Saudi Central Board for Accreditation of Healthcare Institutions laid out safe staffing “nurse to patient ratios” standards in its facilities, the national nursing shortage hinders achieving these standards that led nurses to an excessive workload which is considered as a significant reason for missed nursing care. Therefore, nurses prioritize their patients according to their acuity level, and critical patients usually receive the most attention compared with those who are less critical [36]. Tubbs–Cooley et al. [37] revealed a direct relationship between missed nursing care and nursing workload. Meanwhile, Ball et al. [38] argued that for every rise in the number of patients, the workload of each nurse rises; consequently, the probability of missed nursing care will increase to 10%.

The study also found that approximately 20% of the participants had plans to leave their current position. Such a rate is lower than those in Jordan (32%) [28], Egypt (24.8%) [29], and Italy (35.5%) [39]. Furthermore, the current study revealed that missed nursing care had a significantly positive correlation with their intention to leave their jobs. Missed nursing care is linked to work overload, considering that nurses cannot monitor all patients assigned to them and compel themselves to ration their care [40, 41]. The workload is a main factor that causes missed nursing care [22]. These circumstances usually lead to poor nursing satisfaction, which is also associated with a higher rate of intention to leave [21].

In addition, a significantly negative correlation was noted between missed nursing care and job satisfaction. These variables are strongly related throughout the nursing literature. This finding is supported by a previous study, which recruited 7079 nurses from different countries, including Australia, the US, Turkey, and Iceland and reported that a higher rate of missed nursing care was linked to a lower level of job satisfaction [42]. The level of nursing job satisfaction is an important indicator not only to nurses' physical and psychological health but also to the quality of the healthcare system [43–45]. According to several studies, nursing job satisfaction is directly related to the amount of missed nursing care, considering that staff nurses who reported less missed nursing care often claimed to have a higher level of job satisfaction [23, 31, 46, 47]. Indeed, various reasons can hinder nurses from providing optimum and comprehensive care, leave them frustrated, and eventually, feel dissatisfied with their job. The results of the current study suggest that the level of nurses' job satisfaction and the reasons behind missed nursing care in Jazan hospitals should be explored in depth to mitigate missed nursing care activities and to improve the quality of nursing care provided to patients.

However, the current study has several limitations. It utilized a cross-sectional correlational design, which does not support causation between missed nursing care and staff satisfaction or turnover intentions. The study used an online questionnaire as pandemic precautions recommend which often led to a low response rate. Furthermore, the sample size is relatively small, and it was rendered exclusively in Jazan Region, thereby limiting the generalizability of the study finding among nursing professionals across Saudi Arabia.

## 5. Conclusions

Missed nursing care is evident across all countries, including Saudi Arabia. Nonetheless, the rate of missed nursing care in Saudi Arabia is significantly lower than those in other international and even middle eastern countries. Failure to attend interdisciplinary care conferences, ambulation, and turning and positioning were the most frequently missed nursing care activities. The main reason behind missed nursing care was human resource shortage. Moreover, poor job satisfaction and high intention to leave were directly associated with an increased rate of missed nursing care. Nursing care is holistic and healthcare organizations need to pay more attention to missed nursing care rates and their underlying causes. Optimizing the recruitment process, resource allocation and effective nurses' retention programs are proposed solutions that may be beneficial to mitigate missed nursing care.

## Figures and Tables

**Table 1:** Sample statistics.

Characteristics	Frequency	Percentage
Gender		
Male	66	10.9
Female	538	89.1

Age (years)		
<25	24	4.0
25–34	422	69.9
35–44	128	21.2
45–54	30	5.0

Highest educational attainment		
RN diploma	220	36.4
AND	50	8.3
BSN	310	51.3
MSN or higher	24	4.0

Nursing experience		
≤6 months	14	2.3
>6 months–2 years	102	16.9
>2 years–5 years	132	21.9
>5 years–10 years	182	30.1
>10 years–15 years	114	18.9
>15 years	60	9.9

Overtime duration for the last 3 months		
None	202	33.4
1–12 hours	284	47.0
>12 hours	118	19.5

Plans to leave the current position		
No plans to leave	484	80.1
In the next 6 months	30	5.0
In the following year	90	14.9

How satisfied are you in your current position?		
Very satisfied (*n* (%))	52 (8.6%)	
Satisfied (*n* (%))	84 (13.9%)	
Neutral (*n* (%))	140 (23.2%)	
Dissatisfied (*n* (%))	232 (38.4%)	
Very dissatisfied (*n* (%))	48 (15.9%)	

Independent of your current job, how satisfied are you with being a nurse or a nurse assistant?		
Very satisfied (*n* (%))	12 (2%)	
Satisfied (*n* (%))	42 (7%)	
Neutral (*n* (%))	74 (12.3%)	
Dissatisfied (*n* (%))	208 (34.4%)	
Very dissatisfied (*n* (%))	268 (44.4%)	

How satisfied are you with the level of teamwork on this unit?		
Very satisfied (*n* (%))	12 (2%)	
Satisfied (*n* (%))	58 (9.6%)	
Neutral (*n* (%))	104 (17.2%)	
Dissatisfied (*n* (%))	250 (41.4%)	
Very dissatisfied (*n* (%))	180 (29.8%)	

**Table 2:** Types of missed nursing care.

Items	Never missed	Rarely missed	Occasionally missed	Frequently missed	Always missed	Mean	SD
Attending interdisciplinary care conferences whenever held	369	114	77	44	0	1.66	0.96
	61.1%	18.9%	12.7%	7.3%	0%		

Facilitating ambulation three times per day or as ordered	398	74	92	40	0	1.63	0.97
	65.9%	12.3%	15.2%	6.6%	0%		

Turning and positioning every 2 hours	185	122	76	36	0	1.63	0.92
	61.3%	20.2%	12.6%	6%	0%		

Feeding the patients when the food is still warm	418	82	66	38	0	1.54	0.92
	69.2%	13.6%	10.9%	6.3%	0%		

Teaching the patients about illness, tests, and diagnostic studies	423	96	69	16	0	1.46	0.80
	70.1%	15.9%	11.4%	2.6%	0%		

Providing mouth care	430	104	42	28	0	1.45	0.82
	71.2%	17.2%	7%	4.6%	0%		

Offering emotional support to patients and/or family	219	94	56	16	0	1.42	0.77
	72.5%	15.6%	9.3%	2.6%	0%		

Assisting with toileting needs within 5 minutes of request	436	106	46	16	0	1.41	0.74
	72.2%	17.5%	7.6%	2.6%	0%		

Bathing or providing skin care	454	94	32	24	0	1.38	0.76
	75.2%	15.6%	5.3%	4%	0%		

Responding to call light within 5 minutes	468	84	40	12	0	1.33	0.69
	77.5%	13.9%	6.6%	2%	0%		

Setting up meals for patients who feed themselves	481	72	34	18	0	1.32	0.71
	79.7%	11.9%	5.6%	3%	0%		

Discharge planning and teaching	478	72	40	14	0	1.32	0.70
	79.1%	11.9%	6.6%	2.3%	0%		

Fully documenting all necessary data	482	84	24	14	0	1.29	0.65
	79.8%	13.9%	4%	2.3%	0%		

Responding to PRN medication requests within 15 minutes	496	66	22	20	0	1.28	0.69
	82.1%	10.9%	3.6%	3.3%	0%		

Conducting focused reassessments according to patients' condition	484	80	24	12	0	1.28	0.65
	80.1%	13.2%	4.6%	2%	0%		

Providing skin/wound care	244	39	12	7	0	1.28	0.65
	80.8%	12.9%	4%	2.3%	0%		

Monitoring intake/output	490	74	28	12	0	1.27	0.64
	81.1%	12.3%	4.6%	2%	0%		

Administering medications within 30 minutes before or after the scheduled time	492	140	68	16	0	1.27	0.62
	81.5%	11.6%	5.6%	1.3%	0%		

Assessing the effectiveness of medications	304	66	26	8	0	1.24	0.59
	83.4%	10.9%	4.3%	1.3%	0%		

Performing patient assessments each shift	510	68	12	14	0	1.22	0.59
	84.4%	11.3%	2%	2.3%	0%		

Handwashing	528	50	18	8	0	1.18	0.54
	87.4%	8.3%	3%	1.3%	0%		

Assessing vital signs as ordered	544	40	16	4	0	1.14	0.46
	90.1%	6.6%	2.6%	0.7%	0%		

Bedside glucose monitoring as ordered	548	40	14	2	0	1.12	0.42
	90.7%	6.6%	2.3%	0.3%	0%		

IV, intravenous; PRN, as necessary; SD, standard deviation.

**Table 3:** Reasons for missed nursing care.

Items	NOT a reason for missed care	Minor reason	Moderate reason	Significant reason	Mean	SD
Inadequate number of staff	44	28	98	433	3.53	0.88
	7.2%	4.8%	16.2%	71.8%		

Inadequate number of assistive and/or clerical personnel (nursing assistants, technicians, and unit secretaries)	44	64	128	368	3.36	0.94
	7.3%	10.6%	21.2%	60.9%		

Unexpected rise in patient volume and/or acuity on the unit	70	36	144	354	3.29	1.01
	11.6%	6%	23.8%	58.6%		

Heavy admission and discharge activity	74	74	144	312	3.15	1.05
	12.3%	12.3%	23.8%	51.7%		

Supplies/equipment not functioning properly when needed	74	89	135	306	3.12	1.06
	12.3%	14.7%	22.4%	50.7%		

Unbalanced patient assignments	88	62	148	306	3.11	1.09
	14.6%	10.3%	24.5%	50.7%		

Urgent patient situations (e.g., a worsening condition)	74	80	154	296	3.11	1.05
	12.3%	13.2%	25.5%	49%		

Supplies/equipment not available when needed	78	92	142	292	3.07	1.07
	12.9%	15.2%	23.5%	45.3%		

Lack of backup support from team members	92	88	162	262	2.98	1.09
	15.2%	14.6%	26.8%	43.4%		

Other departments did not provide the care needed (e.g., physical therapy did not ambulate)	88	106	160	250	2.95	1.08
	14.6%	17.5%	26.5%	41.4%		

Tension or communication breakdowns with other ancillary/support departments	100	84	176	244	2.93	1.10
	16.6%	13.9%	29.1%	40.4%		

Tension or communication breakdowns with the medical staff	96	91	192	245	2.93	1.09
	15.9%	15.1%	28.5%	40.5%		

Medications were not available when needed	94	124	126	230	2.91	1.12
	15.6%	20.5%	20.9%	43%		

Tension or communication breakdowns within the nursing team	115	100	146	243	2.85	1.15
	19.1%	16.6%	24.2%	40.1%		

Caregiver off unit or unavailable	122	112	136	234	2.80	1.16
	20.2%	18.5%	22.5%	38.7%		

Inadequate handoff from the previous shift or sending unit	119	123	156	206	2.75	1.13
	19.5%	20.4%	25.8%	34.1%		

Nursing assistant did not communicate that care was not provided	133	112	155	204	2.72	1.15
	21.9%	18.5%	25.7%	33.8%		

**Table 4:** Correlations between missed nursing care, job satisfaction, and intention to leave.

Variables	1	2	3	4	5	6
1. Satisfaction with the current position	1.000					
2. Satisfaction with being a nurse	0.387^*∗∗*^	1.000				
3. Satisfaction with the level of teamwork in the unit	0.494^∗∗^	0.414^∗∗^	1.000			
4. Total satisfaction score	0.818^∗∗^	0.747^∗∗^	0.798^∗∗^	1.000		
5. Intention to leave	−0.212^∗∗^	−0.368^∗∗^	−0.266^∗∗^	−0.353^∗∗^	1.000	
6. Total missed nursing care	−0.199^∗∗^	−0.278^∗∗^	−0.231^∗∗^	0.247^∗∗^	−0.199^∗∗^	1.000

^*∗∗*^ *p* < 0.01.

**Table 5:** Multiple linear regression predicting missed nursing care.

Model summary						
Model	R		R square	Adjusted R square	Std. error of the estimate	
	0.46		0.21	0.20	0.976	
Model		Unstandardized coefficients		Standardized coefficients	t	Sig.
		B	Std. error	Beta		
	(Constant)	59.35	4.21		14.07	0.001
	Age	0.457	0.831	0.026	0.550	0.580
	Gender	−8.592	1.321	−0.245	−6.505	0.060
	Qualification	−0.132	0.468	−0.012	−0.281	0.779
	Level of experience	−0.010	0.427	−0.001	−0.023	0.981
	Adequate staff	−0.762	0.350	−0.087	−2.178	0.030
	Working overtime	−0.038	0.567	−0.002	−.067	0.946
	Turnover intention	2.380	0.622	0.158	3.829	0.001
	Level of satisfaction	−0.864	0.180	−0.198	−4.788	0.001

Model		Sum of squares	Df	Mean square	F	Sig.
	Regression	15.55	10	155.52	16.319	0.001
	Residual	56.51	593	95.30		
	Total	72.06	603			

## Data Availability

The datasets used for this study are available from the corresponding author upon reasonable request.
